# Practical Notes and Formulæ

**Published:** 1881-02-20

**Authors:** 


### PRACTICAL NOTES AND FORMULÆ.

**Diphtheria.**—The following is suggested by a writer (in Lancet and Clinic) as a good treatment in many cases of diphtheria:

First twenty-four hours: Saline cathartic. Topical: tincture iodine all over the cervical region, paint it every four or six hours. Locally:

℞ Hyposulphite sodæ........................................ ℥ i

Aq. menth. pip................................................ ℥ vii

Bro. ammonium................................................ ʒ iv Mix.

Gargle every two hours and take one teaspoonful at same time. Constitutional treatment:

℞ Carb. ammonia................................................ ℥ ss

Acid acet. dil................................................ f℥ i Mix.

After effervescing add:

Tinct. ferri chloridi................................................ ℥ i
Glycerin pure,
Aq. menth. pip................................................ aa qs
Ft. oz. viij................................................ (f℥ viij)

S. One tealpoonful every two hours.

At the expiration of forty-eight hours we use the iron mixture every two hours, alternating with whisky and quinine, and continue the latter until our patient is perfectly restored. Topical applications are of but little consequence after two or three days. In place of the iodine some oleaginous mixture should be applied to the neck for two or three days.

**A Vehicle for Salicylic Acid.**—A pleasant and agreeable method of administering salicylic acid is as follows: Take Oswego corn-starch one tablespoonful, to be thoroughly rubbed up in several ounces of cold water. Add a quart of milk, set on the fire, and stir until the mixture has boiled sufficiently to become homogeneous. The addition of sugar and essence of vanilla or lemon will give a delicious blanc-mange. Twenty grains of the salicylic acid can be rubbed up in a mortar with a cupful of the blanc-mange, which may be eaten warm or cold. The acid taste is entirely disguised, and a medicine irritating to a healty stomach can be safely administered in combination with a nutritious but light food to such patients as are in need thereof.—Dyer, in *Louisville News*, Oct. 8.

**Esmarck's Caustic Powder.**—

Arsenious acid................................................ 15 grains.
Sulphate of morphia................................................ 15 "
Calomel................................................ 2 drachms.
Powdered gum arabic................................................ 12 "

Mix. This is sometimes called painless caustic powder, although the propriety of the name is doubted by some.—*Drug. Cir.*

**Bismuth Hair Dye.**—

No. 1.

| | |
|---|---|
| Citrate of bismuth | 1 ounce. |
| Rose water | 2 ounces. |
| Distilled water | 2 " |
| Alcohol | 5 drachms. |
| Ammonia, sufficient. | |

No. 2.

| | |
|---|---|
| Hydrosulphate of soda | 12 drachms. |
| Distilled water | 4 ounces. |

Each solution is to be applied separately, the No. 1 first, and No. 2 when this has dried thoroughly. To insure success, the hair must previously be well freed from all oily substances, natural or added, by means of some of the weak alkaline solutions commonly known as shampoos. The hair, being then rapidly dried, is ready to receive the dye. These remarks apply to all hair colorings.—*Drug. Cir.*

**Salicylic Acid as a Foot Powder.**—As a protection to the feet, in the Russian army, sylicylic acid is used. It is in the shape of a powder, and is a great preventive against perspiring and sore feet.

COMPOSITION.

| | |
|---|---|
| Acid salicylic | 3 parts. |
| Amylum | 10 " |
| Powder of taltum | 87 " |

It is applied dry; on a march daily; in garrison, every two or three days. It takes off the irritating influence of the perspiration of the feet, and prevents, in consequence, the soreness.

In the Italian army aniseed is similarly used in hot weather.—*Med. and Surg. Rep.*

**Furniture Polish.**—

| | |
|---|---|
| Linseed oil | 2 pints. |
| Alcohol | ½ pint. |
| Vinegar | ½ " |
| Butter of antimony | 2 ounces. |
| Spirit of turpentine | ½ pint. |

Shake well before using, and apply with a woolen rubber.—*Druggists' Circular.*

**Hair Dye with One Preperation.**—The following can be used for the whiskers as well as for the hair. None of them is, or can be, patented:

| | |
|---|---|
| Nitrate of silver | 1 ounce. |
| Distilled water | 8 ounces. |
| Water of ammonia, sufficient. | |

Dissolve the silver salt in water, and add ammonia to the solution until the precipitate formed at first is redissolved. This gives a black dye. To obtain a brown color, increase the proportion of ammonia and water.

**Peptized Milk as Food for Infants and Invalids.**—Nunn recommends the following modes of preparing this valuable food: Take one pint of milk at 80°F., add a teaspoonful of rennet solution to 10 grains of pepsin, and keep the mixture at 80°F. When coagulation is complete, but before the whey has begun to separate, beat the whole up smooth with a whisk or beater, and pass through a fine milk-strainer to insure the minute division of the curd. This preparation appears to keep equally as well, or better, than raw milk, remaining apparently unchanged for twenty-four hours if kept cool. Dilute and sweeten for feeding as usual.

By this method coagulation is complete, and no further change of that nature is requisite, the weakened stomach of the invalid receives the necessary nutriment, carrying with it its own digestive principle.—*Buffalo Med. and Surg. Jour.*, Dec., 1880.

**Effervescent Beverages.**—A correspondent of the London *Chemist and Druggist* supplies the following formulæ:

GINGER BEER.

| | |
|---|---|
| Brown sugar | 2 pounds. |
| Boiling water | 2 gallons. |
| Cream of tartar | 1 ounce. |
| Bruised ginger root | 2 ounces. |

Infuse the ginger in boiling water, add your sugar and cream of tartar; when lukewarm strain; then add half pint good yeast. Let it stand all night, then bottle; if you desire, you can add one lemon and the white of an egg to fine it.

LEMON BEER.

| | |
|---|---|
| Boiling water | 1 gallon. |
| Lemon sliced | 1 |
| Ginger, bruised | 1 ounce. |
| Yeast, | 1 teacupful. |
| Sugar, | 1 pound. |

Let it stand 12 to 20 hours, and it is ready to be bottled.

HOP BEER.

| | |
|---|---|
| Water | 5 quarts. |
| Hops | 6 ounces. |

Boil three hours, strain the liquor, add

| | |
|---|---|
| Water | 5 quarts. |
| Bruised ginger | 4 ounces. |

And boil a little longer, strain, and add 4 pounds of sugar, and when milk-warm, 1 pint of yeast. Let it ferment; in 24 hours it is ready for bottling.—*Druggist's Circular.*

**Nervine.**—

| | |
|---|---|
| ℞ Tinc. hops, | |
| Tinc. valerian, | |
| Camphor water | aa ℥ iij. |
| McMun's elix. opii | ʒ ij. M. |

Dose, tablespoonful. Excellent anodyne in nervous conditions, or to promote rest in cases of insomnia.

**Bronchitis.**—Dr. Fothergill, of London, recommends the following cough mixture in the obstinate cough of bronchitis:

| | | |
|---|---|---|
| ℞ | Chloroform, | gtt xx. |
| | Hydrobromic acid | ʒ ss. |
| | Syr. of squills, | ʒj. |
| | Water to make | ℥j. |

Dose—One to two tablespoonfuls three to four times a day.

In Bellevue Hospital the following cough mixture is used:

| | | |
|---|---|---|
| ℞ | Ammon. carb | ½ drachm. |
| | Fluid ext. senega | — |
| | Fluid ext. squill | aa 1 drachm. |
| | Paregoric | 6 drachms. |
| | Water, | ½ ounce. |
| | Syr. tolu q. s. to make | 4 ounces. |

Dose—One tablespoonful.

**Remedies for Chilblains.**—The following formula for Dr. Valentine Mott's Remedy is given in the Proceedings of the Medical Society of the County of Kings:

| | | |
|---|---|---|
| ℞ | Beef's gall | 4 ounces. |
| | Ol. terebinth | 4 " |
| | Spts. vini rect., 90 per cent | 1½ " |
| | Tinct. opii | 1 " |

Another formula for the same affection is—

| | | |
|---|---|---|
| ℞ | Beef brine | 1 pint. |
| | Potassæ nitratis | 2 drachms. |
| | Aquæ ammoniæ | 3 ounces. |

—*Druggists' Circular.*

**For Asthma.**—

| | | |
|---|---|---|
| ℞ | Potassii iodid | 2 drachms. |
| | Morphæ sulphatis | ¾ grain. |
| | Tinc. squills, | |
| | Tinc. lobelia, | |
| | Syrup | aa 1 ounce. |

M. A tablespoonful three times a day in asthma with emphysema and chronic bronchitis.

**Treatment of Choleraic Diarrhœa by the Hypodermic Injection of Morphia.**—Mr. W. Hardman says (*Lancet*, vol. ii., 1880, p. 538) that choleraic diarrhœa can always be immediately stopped by the administration of morphia hypodermically. If severe diarrhœa has persisted over two hours in spite of the administration of morphia and opium, hypodermic injection of morphia should be at once resorted to. The treatment is absolutely free from danger, even if albuminuria or temporary suppression of urine be present. In severe cases obstinate vomiting may persist for twelve to forty-eight hours after the purging is stopped; but this need not occasion any anxiety; it is the purging that kills.—*Times.*

